# Modeling and optimization method of an indirectly irradiated solar receiver

**DOI:** 10.1016/j.mex.2018.12.006

**Published:** 2018-12-17

**Authors:** Baye A. Ndiogou, Ababacar Thiam, Cheikh Mbow, Mohamed Izzedine S. Adjibade, Vincent Sambou

**Affiliations:** a Laboratoire d’Energétique Appliquée, Ecole Supérieure Polytechnique, Université Cheikh Anta Diop de Dakar, PO:5085, Dakar-Fann, Senegal; b Departement de Physique, Université Alioune Diop de Bambey, Senegal; c Faculté des Sciences et Techniques, Université Cheikh Anta Diop de Dakar, Senegal

**Keywords:** Coupling net-radiation method using infinitesimals areas and CFD code an, Coupling of the Kriging surface response method and the MOGA, CSP, Air solar receiver, Net-radiation method, CFD modeling, Response Surface Method optimization, MOGA

## Abstract

This work presents the modeling and optimization of an indirectly irradiated solar receiver. A numerical model of the cavity-absorber block is put forward with the coupling of the net-radiation method using infinitesimal areas and a CFD code. An iterative method with a relaxation factor made it possible to obtain the temperature distribution and the developed code was implemented in the form of UDF and used as boundary conditions in the CFD model of the absorber to simulate the flow of air and heat transfer. The good ability of the receiver to transfer heat to the fluid is proved with a 92% thermal efficiency obtained. Then the combination of the Kriging surface response method and the MOGA allowed the mathematical optimization of the receiver. The multi-objective optimization made it possible to obtain 3 candidates giving the best combinations of design parameters from the fixed objectives.

Three bullet points, highlighting the customization of the procedure.

• A practical analysis using the net-radiation method using infinitesimal areas is applied for cavity radiative exchange model.
• The code developed for the cavity is implemented in the boundary conditions at the level of the ANSYS Fluent CFD model allowing the simulation of the conjugated transfers within the absorber.
• The optimization method proposed is the combination of the Kriging surface response method for quantitative and qualitative analysis of the design parameters and MOGA to obtain different combinations seeking to maximize or to minimize the chosen parameters.

## Graphical abstract

**Figure:**
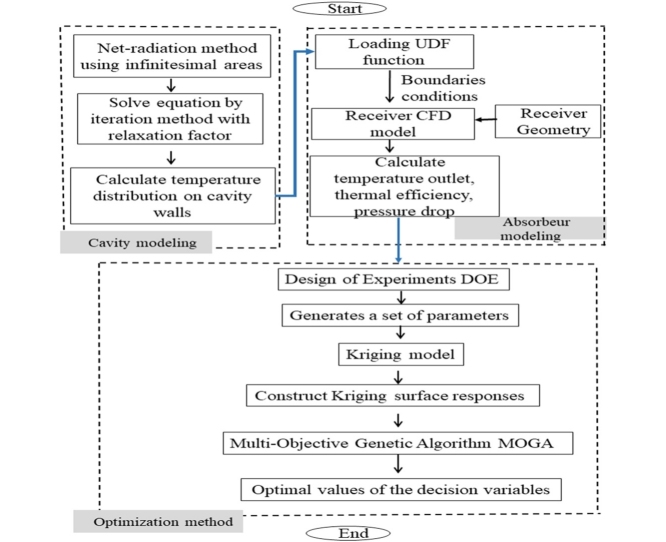

**Specifications table**

**Table:** 

Subject area	• *Energy* • *Computer Science*
More speciﬁc subject area	Central receiver systems
Method name	- Coupling net-radiation method using infinitesimals areas and CFD code an - Coupling of the Kriging surface response method and the MOGA
Name and reference of original method	Net-radiation method using infinitesimals areas is developed in John R. Howell, M. Pinar Menguc, and Robert Siegel. Thermal Radiation Heat Transfer. 6th Edition, Taylor and Francis, 2015. Combination of CFD and optimization models is developed by M.A. Moghimi, K.J. Craig, J.P. Meyer “Optimization of a trapezoidal cavity absorber for the Linear Fresnel Reﬂector” Solar Energy 119 (2015) 343–361.
Resource availability	Visual Studio 2013, ANSYS Workbench 16.0 package

## Method details

### Introduction

**Fig. 1:**
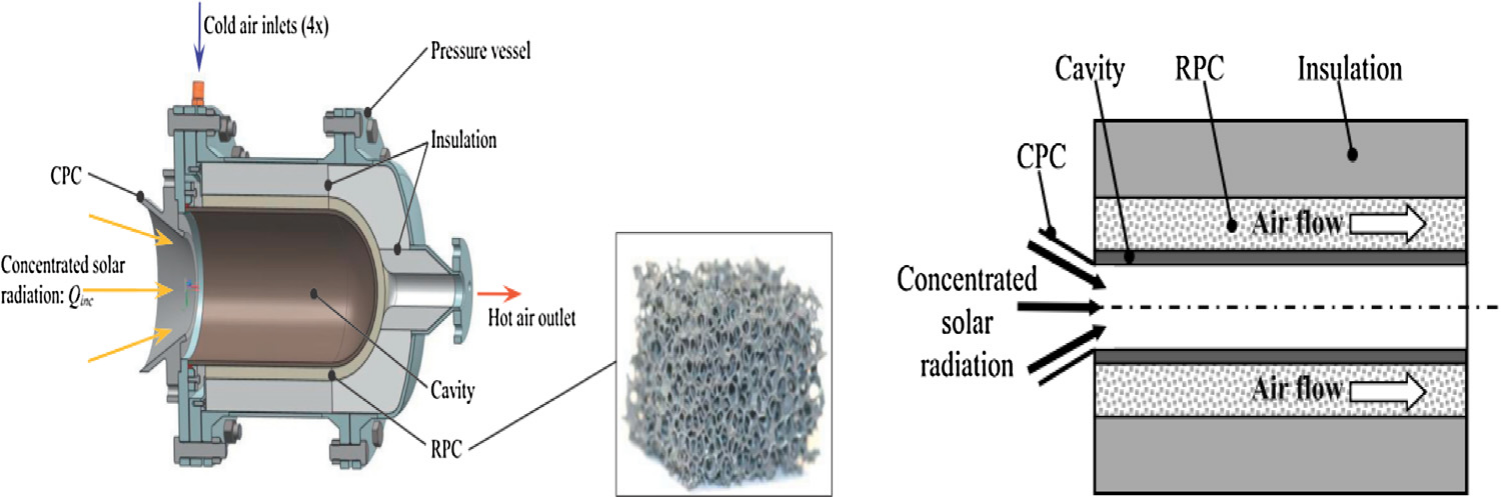
Presentation of the receiver model.

The receiver model is made up by the CPC (Compound Parabolic Collector), which is the second concentrator, incorporated in the front to minimize radiation losses; the horizontal axis (Oz)  and radius $R_{c}$ cavity which absorbs the concentrated solar flux and transmits it by conduction to the RPC; the reticulated porous ceramic RPC plays the role of absorber and is made of a ceramic foam; the outer cylinder acts as an insulator. For the cavity and the porous matrix silicon carbide SiC is considered and the metal alloy Al_2_O_3_-SiO_2_ serves as an outer cylinder (Fig. 1) [1].

The critical component is the ceramic cavity because it operates in severe conditions with the reception of concentrated solar flux. Its configuration leads to efficient capture of solar rays through multiple internal reflections. This implies a non-uniform distribution of temperature on interior surfaces [1,2]. The precise simulation of the process of solar radiation transfer and solar flux distribution could be the essential foundation for the design and optimization of the receiver [3]. Various works have indeed focused on this problem. Many of them used the MCRT method. Wang et al [4] used a Ray Tracing method with the FRED tool to obtain the boundary conditions of the absorber. The model coupled with an analytical method of heat transfer made it possible to find a possible cavity receptor design with a relatively uniform surface temperature. Fang et al [5] proposed the Monte-Carlo method for calculating the surface heat flux inside the cavity. Nevertheless, the description of the radiation heat transfer in the receiver is often not complete by the Monte Carlo method because it only considers the activity of the incident rays, without including the infrared radiation of the interior surfaces of the cavity. In addition, in order to obtain high-precision results, the emission and absorption of tens of thousands of rays need to be tracked during the simulation of the radiative transfers of the cavity using Monte Carlo method that could cost a lot of time and memory [6].

This has led many authors to the net-radiation method. Deng et al [6] proposed a radiation heat transfer model based on the net-radiation method and the thermal equilibrium principle in this study. The method on the basis of form factors is supposed to overcome the disadvantages of the Monte Carlo method on the huge amount in time and memory when calculating radiation in a cavity receiver. Wang et al [3] used Gebhart's method to simulate multiple reflections inside a cavity. This method is proposed to improve the net-radiation method. The results showed that the cavity effect could to a certain extent homogenize the concentrated solar flux distributed on the inner surfaces and substantially reduce the reflection losses of the receiver.

In this paper a practical analysis using the net-radiation method using infinitesimal areas is proposed. This choice makes it possible to take into account the large variations of the temperature *T*, the net-radiation flux density *q*, the irradiation *G* and the radiosity *J* in the calculations [7]. This is explained by the possibility of making several calculations in which smaller elementary surfaces are successively used (and therefore more simultaneous equations) until the results do not change significantly when the areas decrease. The proposed method also allows for a simpler approach to calculating shape factors within the cylindrical cavity.

Once the concentrated solar flux is absorbed by the cavity, the heat is transmitted to the absorber made of a ceramic foam. A mathematical formulation based on the representative elementary volume is used to overcome the discontinuity of the porous medium in the study of fluid flow coupled with heat transfer. It is a method of associating a mathematical point with the properties of a sufficiently representative volume to define or measure the average properties of the volume [8,9]. These two mathematical models will form the basis of a numerical model of the cavity-absorber unit. Indeed an iterative method with a relaxation factor is advanced to obtain the temperature distribution thanks to the numerical resolution of the integral equations giving the radiosity. The code developed is then implemented in the boundary conditions at the level of the ANSYS Fluent CFD model allowing the simulation of the conjugated transfers within the absorber. It will therefore be a question of developing a model based on the coupling of the net-radiation method using infinitesimal areas and a CFD code for the analysis of heat transfer and air flow within the receiver.

Higher temperature outlet and thermal efficiency of solar receiver can directly affect the service life and efficiency of the entire solar power system [2,10]. Many researchers investigated various methods to improve the efﬁciency of the thermal receiver [11]. Several studies have shown that the receiver performance depends on both geometric and physical parameters [[12], [13], [14], [15], [16], [17]]. Many of them study the influence of the parameters influencing individually. Pozivil et al [13] tested the influence of different absorber configurations with different porosities on thermal efficiency. The influences of pressure and mass flow on thermal efficiencies and outlet temperatures were also evaluated. However this approach does not allow to take into account objective functions being in conflict. The consideration of an optimization providing an ideal combination of all the parameters is very interesting. Multi objective optimization can provide ideal combinations of input and operating parameters that can satisfy multiple objectives to obtain a compromise between the parameters that are in mutual conflict. This makes it possible to adapt the receiver to any power or any temperature outlet of the desired fluid, thus allowing a better coupling between the receiver and the various systems of the plant. Multi objective optimization models were proposed in the literature. Moghimi and al [18] uses MOGA combined with the Kriging surface response method to ﬁnd an optimal design for a set of operating conditions. The objective functions that are used to judge the performance of a 2-D cavity are the combined heat loss through convection, conduction and radiation, as well as a wind resistance area. In this paper the effect of absorbed irradiation is introduced in the form of an outer surface of pipe temperature. Seven geometrical parameters are used as design variables. Based on a sample set requiring 79 CFD simulations, a global utopia point is found that minimizes both objectives. Pu and al [19] have also proposed a MOGA combined with the Kriging surface response method to optimize the design parameters of the heat exchanger for a geothermal heat pump. The effect of design parameters, including inlet velocity, inlet temperature, U-tube diameter, hole diameter, and tube spacing on entropy number and factor integrated evaluation were analyzed. The results prove that the optimization method is veriﬁed to be successful. To the best of the authors' knowledge no work has been published in the literature using this optimization method on an indirectly irradiated receiver.

The optimization strategy proposed in this work is the combination of the Kriging surface response method and MOGA. This coupling makes it possible to obtain combinations which can satisfy at the same time several objectives [[18], [19], [20], [21]]. This is due to the fact that the response surface analysis makes it possible to identify qualitatively and quantitatively the dependence of the results with respect to multiple parameters. Then the MOGA makes it possible to obtain different combinations seeking to maximize or to minimize the chosen parameters. This optimization model can provide results that can satisfy multiple objectives to achieve a compromise between the parameters that are in conflict with each other. This makes it possible to adapt the receiver to any power or any output temperature of the desired fluid, thus allowing a better coupling between the receiver and the various systems of the plant.

The objective of this work is therefore first of all to develop an innovative numerical model that is not very greedy in terms of calculation time and resources to predict the behavior and performance of the receiver. And secondly, this work proposes an optimization methodology that can give the best combinations of operating parameters to maximize the amount of thermal energy and thermal potential. The combination of these two models aims to obtain a solar receiver that can properly and efficiently feed the gas turbine.

### Mathematical model

In order to develop mathematical models governing the function of the receiver, a thorough understanding of the heat transfer and air flow mechanisms is required. For the receiver modeling, the part that will be taken into account in this work is that immediately following the CPC. It will therefore be a question of treating the radiative transfers at the level of the cavity by neglecting the losses by natural convection, and the results obtained will be used as boundary conditions for the absorber CFD model.

#### Modeling radiative transfers within the cavity

The solar radiation transfer process in the SPT can be approximately divided into two sub-processes: the transfer process in the solar ﬁeld, and the multiple-reﬂection process inside the cavity [2,10]. In this work the second process is considered. A complex radiative exchange takes place inside the cavity. When the radiation leaves a surface, it is partially reflected and absorbed several times within the enclosure with each contact with a surface. It is therefore a very complicated task to follow the radiation as it undergoes this process. A practical analysis can be formulated with the net-radiation method using infinitesimal areas [7].

In the case where the enclosure is divided into finite areas, the accuracy of the results is limited by the assumption that the temperature and energy arriving at and leaving each surface are uniform over that surface. If the quantities are not uniform over part of the enclosure boundary, that part must be subdivided until the variation between each elementary surface of the analysis is significantly large. Several calculations can be made in which successively smaller elementary surfaces (and hence more simultaneous equations) are used until the results no longer change significantly when the area sizes decrease. This makes it possible to consider the large variations of the quantities *T*, *q*, *G* and *J* for the calculations [7].

Assuming the following simplifying assumptions:

- The cavity is considered as an enclosure of N finite areas which are further subdivided into differential elements.
- The air in the cavity is a non-absorbing gas
- The surfaces of the cavity are diffuse and gray
- For simplicity the radiative properties are independent from the temperature.

The net-radiation flux density on the wall ${\;q}_{r,w}\left(R_{i},z\right)$, which is the difference between the outgoing flux of the radiation leaving the wall ${\;J}_{w}\left(R_{i},z\right)$, and the illumination from the other surfaces $G_{w}\left(R_{i},z\right)$ is written

(1) $$q_{r,\text{w}}\left(R_{i},z\right)=J_{w}\left(R_{i},z\right)-G_{w}\left(R_{i},z\right)$$

The outgoing flux is composed of emitted and reflected energy

(2) $$J_{w}\left(R_{i},z\right)=\varepsilon_{w}\;\sigma T_{w}^{4}\left(R_{i}\right)+(1-\varepsilon_{w}\;)G_{w}\left(R_{i},z\right)$$

The incoming flux is composed of portions of the outgoing fluxes from the other areas of the enclosure

(3) $$G_{w}\left(R_{i},z\right)=\sum_{l}^{L}\int_{S_{l}}^{}J_{l}.{K\left({\vec{r}}_{w};{\vec{r}}_{l}\right).dS}_{l}$$

Eq. (2) provide two expressions for${\;G}_{w}\left(R_{i},z\right)$. These are each substituted into Eq. (1)

(4) $$q_{r,\text{w}}(R_{i},z)=\frac{\varepsilon_{w}}{1-\varepsilon_{w}}[\sigma T_{w}^{4}\left(R_{i}\right)-J_{w}\left(R_{i}\right)]$$

(5) $$q_{r,\text{w}}(R_{i},z)=J_{w}(R_{i})\;-\sum_{l}^{L}\int_{S_{l}}^{}J_{l}.{K\left({\vec{r}}_{w};{\vec{r}}_{l}\right).dS}_{l}$$

With ${\vec{r}}_{p}$ and ${\vec{r}}_{l}$ as the positions of the elementary surfaces $\;{dS}_{w\;}$ and $\;{dS}_{l}$, and $\;K\left({\vec{r}}_{w};{\vec{r}}_{l}\right)$, the Kernel is defined by

(6) $$K\left({\vec{r}}_{w};{\vec{r}}_{l}\right)=\frac{\left({\vec{n}}_{w}.{\vec{r}}_{w,l}\right).\left({\vec{n}}_{l}.{\vec{r}}_{l,w}\right)}{\pi .{\left|{\vec{r}}_{w,l}\right|}^{4}}$$

To determine the temperature by the combination of Eqs. (4) and (5), it is necessary to solve the equation of radiosity given by

(7) $$J_{w}(R_{i},z)\;=\varepsilon_{w}\sigma T_{w}^{4}-\left(1-\varepsilon_{w}\right)\sum_{l=1}^{L}\int_{S_{l}}^{}J_{l}.{K\left({\vec{r}}_{w};{\vec{r}}_{l}\right).dS}_{l}$$

Considering the aperture and outside surfaces as black surfaces at constant temperatures $T_{a}$ and $\;T_{s}$, the following integral equation is obtained

(8) $$J_{w}\left(R_{i},z\right)=\varepsilon_{w}\sigma T_{w}^{4}-\left(1-\varepsilon_{w}\right)\sigma .\int_{S_{e}}^{}T_{a}^{4}\left(r,z=0\right).K\left({\vec{r}}_{w};{\vec{r}}_{e}\right).\;dS-\left(1-\varepsilon_{w}\right)\sigma .\int_{S_{s}}^{}T_{s}^{4}\left(r,z=L\right).{K\left({\vec{r}}_{w};{\vec{r}}_{s}\right).dS}_{s}T-\;\left(1-\varepsilon_{w}\right)\int_{S_{p\text{'}}}^{}J_{p}.{K\left({\vec{r}}_{w};{\vec{r}}_{w\text{'}}\right).dS}_{w\text{'}}$$

The last term represents the radiative inter-wall contribution.

After the *J* is found from these simultaneous integral equations, Eq. (3) is applied to determine the unknown T distributions

(9) $$\sigma T_{w}^{4}\left(R_{i}\right)=\frac{1-\varepsilon_{w}}{\varepsilon_{w}}q_{r,\text{w}}\left(R_{i},z\right)+J_{w}\left(R_{i}\right)$$

#### Modeling of air flow and heat transfers within the absorber

At the absorber level there is a forced convection flow in an annular space filled with a porous material delimited by two concentric cylinders of horizontal axis of length L. At the entrances, air is injected with a mass flow rate $\;\dot{m}$ and temperature *T_a_*. For the two-dimensional model, two air inlets and one outlet are considered. To overcome the discontinuity of the porous medium that constitutes the absorber, the notion of the representative elementary volume (R.E.V.) is used as a mathematical framework for writing equations. It is a method of associating a mathematical point with the properties of a sufficiently representative volume to define or measure the average properties of the volume [8,20]. The following assumptions are adopted

- The porous medium is homogeneous and isotropic
- The scheme is permanent
- The flow in the channel is incompressible, turbulent and two-dimensional
- The fluid is Newtonian
- The viscous dissipation in the energy equation as well as the work of the pressure forces are negligible
- Hydrodynamic and thermal boundary layer hypotheses are valid

Under the conditions of validity of the Forchheimer-Wooding model, the equations of continuity, momentum and energy in cylindrical coordinates are written as follows:

(10) $$\frac{\partial \left(\rho u_{z}\right)}{\partial u_{r}}+\frac{\partial \left(\rho u_{r}\right)}{\partial u_{z}}=0$$

(11) $$\frac{1}{\varphi^{2}}\left(u_{z}\frac{\partial \left(\rho u_{z}\right)}{\partial r}+u_{r}\frac{\partial \left(\rho u_{r}\right)}{\partial z}\right)=-\frac{\partial P}{\partial r}+\mu \left\{\frac{\partial }{\partial r}\left[\frac{1}{r}\frac{\partial }{\partial r}(ru_{r})\right]+\frac{\partial u_{r}}{\partial z^{2}}\right\}+\left[-\frac{\mu }{K}-F\rho \left|u\right|\right].u_{r}$$

(12) $$\frac{1}{\varphi^{2}}\left(u_{z}\frac{\partial \left(\rho u_{z}\right)}{\partial r}+u_{r}\frac{\partial \left(\rho u_{r}\right)}{\partial z}\right)=-\frac{\partial P}{\partial z}+\mu \left\{\frac{1}{r}\frac{\partial }{\partial r}\left[r\frac{\partial u_{z}}{\partial r}\right]+\frac{\partial u_{z}}{\partial z^{2}}\right\}+\left[-\frac{\mu }{K}-F\rho \left|u\right|\right].u_{z}$$

(13) $$u_{r}\frac{\partial \left(\rho c_{p}T\right)}{\partial r}+u_{z}\frac{\partial \left(\rho c_{p}T\right)}{\partial z}=k_{eff}\left\{\frac{1}{r}\frac{\partial }{\partial r}\left[r\frac{\partial T}{\partial r}\right]+\frac{\partial T}{\partial z^{2}}\right\}-div(\vec{q_{r})}$$

$\frac{\mu }{K}u_{z}$: Darcy’s term

$\left[-\frac{\mu }{K}-F\rho \left|u\right|\right].u_{r}$: Forchheimer's term.

$q_{r\;}$ is the radiative flux density vector.

$k_{eff}$ and $\mu_{eff}$ are the effective thermal conductivity and dynamic viscosity of the porous medium.

The radiative transfer incorporated through the specter can be reduced to the following quasi stationary equation

(14) $$\frac{1}{\alpha }div\left[\vec{e}.L(M,\vec{e})\right]+L\left(M,\vec{e}\right)=\frac{n^{2}.\sigma .T^{4}}{\pi }$$

The divergence of the radiative flux density vector is given by

(15) $$div\left(\vec{q_{r}}\right)=4.n^{2}.\sigma .\alpha_{w}.T^{4}-\int_{\text{Ω}=4\pi }^{}\left[\int_{v}^{}{\;\alpha }_{v}L\left(M,\vec{e}\right).dV\right]d\text{Ω}$$

The thermal efficiency is given by

(16) $$\eta_{thermal}=\dot{m.}(h_{outlet}-h_{inlet})/q_{incident}$$

Where $\;h_{outlet}$ and $h_{inlet}$ are the enthalpies of the fluid respectively at outlet and inlet. And $q_{incident}$ incident flux.

### Numerical model

The equations obtained at the level of the cavity and the absorber being strongly non-linear, a numerical resolution is necessary. The strategy used in this paper consists first of all in solving the integral equations, which makes it possible to obtain the distribution throughout the cavity. Then these results are loaded into CFD ANSYS Fluent model under boundary conditions for the simulation of conjugate transfers within the absorber. This makes it possible to obtain a numerical model of the bloc cavity-absorber.

#### Principle of the integral equation solving method

It is a question of solving the integral equation of Fredholm, by means of an iterative process with a relaxation factor

(17) $$J_{w}\left(z\right)=\;J\left(z\right)\;et\;C.\int_{0}^{L}J_{w}\left(z^{\text{'}}\right).K\left(z;z^{\text{'}}\right).dz\text{'}=F(z)$$

With

(18) $$f\left(z\right)=\varepsilon_{w}\sigma T_{w}^{4}-\left(1-\varepsilon_{w}\right)\sigma .T_{a}^{4}.\int_{S_{e}}^{}K\left({\vec{r}}_{w};{\vec{r}}_{e}\right).\;dS-\left(1-\varepsilon_{w}\right)\sigma .T_{s}^{4}.\int_{S_{s}}^{}{K\left({\vec{r}}_{w};{\vec{r}}_{s}\right).dS}_{s}$$

And

(19) $$C=-\left(1-\varepsilon_{w}\right)$$

The following expression will be obtained

(20) $$J\left(z\right)=J_{w}\left(z\right)\;et\;F(z)=C.\int_{0}^{L}J_{w}\left(z^{\text{'}}\right).K\left(z;z^{\text{'}}\right).dz\text{'}$$

The segment [0; *L*] is returned to m nodes ${\;z}_{i}$, regularly spaced by a constant pitch Δz.

Then this expressions are submitted

(21) $$z=\Delta z\text{*}\left(i-1\right)\;;\;J\left(z\right)=J_{i}\;;f\left(z\right)=\;f_{i};\;F\left(z\right)=\;F_{i}$$

The relaxation parameter is ω and the stop criterion ${\;C}_{stop}$.

The set of resolution steps can be summarized by the following flowchart

**Fig. 2:**
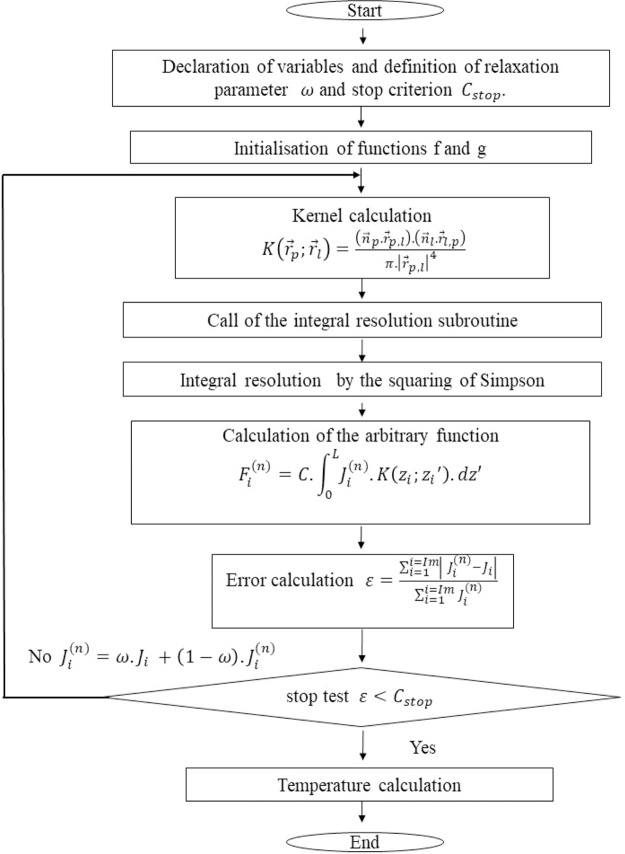
Flowchart of the digital procedure.

The results obtained with the code inspired by this flowchart make it possible to obtain the temperature distribution throughout the cavity (Fig. 2).

#### CFD model of the absorber

The ANSYS Workbench 16.0 package is used for the entire numerical procedure including ANSYS Spaceclaim geometry, ANSYS meshing mesh and CFD modeling with ANSYS Fluent. For this study a receiver with the optimized dimensions (Table 1) is chosen [12]

**Table 1:** Dimensions of the receiver.

Parameters	Dimensions (m)
Cavity diameter	0.5
Aperture diameter	0.25
Cavity thickness	0.02
RPC thickness	0.01
Insulator thickness	0.1
Cavity Length	0.5

**Fig. 3:**
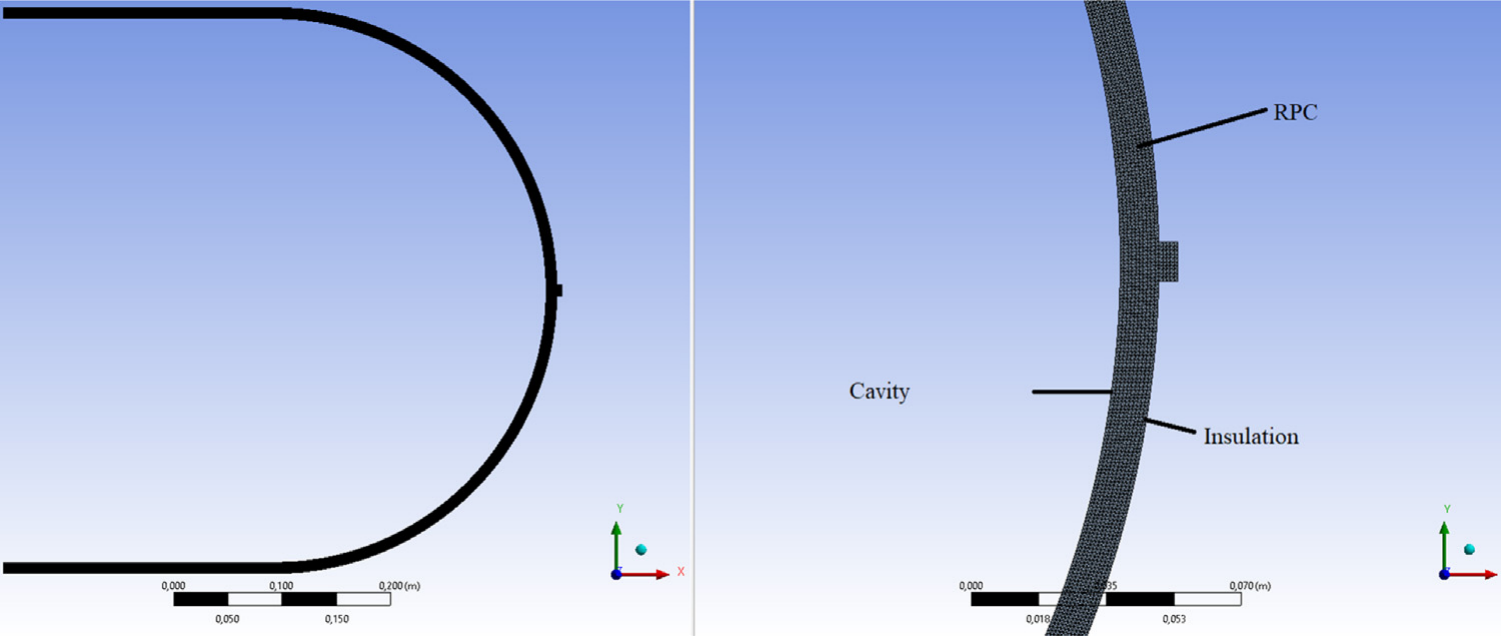
Computational domain of the model.

The mesh meshing package ANSYS Meshing 16 was used to generate the mesh throughout the process of numerical simulation and optimization. A uniform 2D triangular mesh is applied at the geometry level. A maximum size of the elements is set at 0.001 m with a curvature angle of 10°. A total of 26,120 elements is generated in the computational domain (Fig. 3). In order to provide more freedom for automatic mesh generation during the optimization process, the behavior of these face size elements has been set to "Soft". CFD, Fluent and 100 have been selected as "Physics Preferences", "Solver Preference" and "Relevance" in the mesh tools [18,22].

**Fig. 4:**
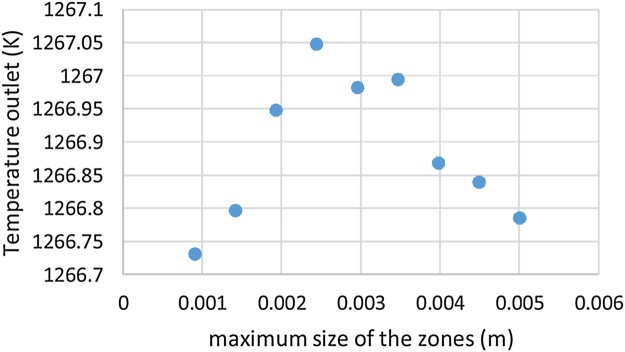
Outlet temperature according to the maximum size of the mesh areas.

The Fig. 4. Shows the grid independence test with the variation of the outlet temperature of the fluid as a function of the maximum size of the zones. The results shows that the deviation of the temperature outlet versus the maximum size of the zones were less than 1%.

The conservation equations of mass, momentum and energy are discretized by the finite volume method. The coupling between speed and pressure is based on the SIMPLEC algorithm. The turbulent k-epsilon model is used with the ” Realizable ” option. For wall treatment the ” Enhanced ” option is used for simulating forced convection inside the absorber. The model of thermal radiation used is the discrete ordinate method. S. Mey et al [23] have shown that this method is perfectly suited for solving the equation of radiative transfers within this type of absorber.

The optical properties of the ceramic foam, namely the absorption and destruction diffusion coefficients, were obtained using the formulas of Zhiyong Wu and al. [24].

(22) $$D_{v}=1.5\left(2-\alpha \right)\frac{\left(1-\varphi \right)}{d}$$

(23) $$\alpha_{v}=1.5\alpha \frac{1-\varphi }{d}$$

(24) $$\beta =D_{v}+\alpha_{v}$$

The thermal-physical properties of air dependent on temperature are given by the following correlations [4].

(25) $$C_{p}=1060+0.449T+1.14.10^{-3}T^{2}-8.10^{-7}T^{3}+1.93.10^{-10}T^{4}$$

(26) $$\mu =1.13.10^{-6}+7.06.10^{-8}T-4.87.10^{-11}T^{2}+2.66.10^{-14}T^{3}-6.12.10^{-18}T^{4}$$

(27) $$k_{f}=-3.94.10^{-4}+1.02.10^{-4}T-4.86.10^{-8}T^{2}+1.52.10^{-11}T^{3}-6.12.10^{-18}T^{4}$$

The properties of the silicon carbide are given by the following relations [23].

(28) $$C_{pSiC}=198.3+2.31T-2.193T^{2}-1.032.10^{-6}T^{3}+1.93.10^{-10}T^{4}$$

(29) $$k_{SiC}=203.1-0.4176T+4.365.10^{-4}T^{2}+2.2.10^{-7}T^{3}+4.232.10^{-11}T^{4}$$

Boundary conditions: Coupling between the net-radiation method and the CFD code.

The code developed to obtain the temperature distribution at the cavity is written in C language and implemented in ANSYS Fluent as a user-defined function (UDF). A user-defined function is a function provided by the user to interact with the CFD solver by providing external inputs, such as boundary conditions, material properties, or source terms. In this work, it is necessary to allow the solver to take into account the complex phenomena that occur at the level of the cavity. The code is first compiled and then loaded as a library and can be used at the boundary conditions [25]. Table 2 gives the main operating parameters

**Table 2:** Mains operating parameters.

Inlet mass flow (kg/s)	0.1
Porosity	0.85
Inlet temperature (K)	473
Operating pressure (Pa)	500,000

### Method validation

Some experimental and validated works are available from the literature. Refs. [[12], [13], [14]] have been chosen and deeply investigated in order to validate the current work. The detailed information of the validation conditions is as follows:

#### Operating conditions

• Case 1

Hishier et al [12] developed a model of the same type of receiver validated by experimentation on a 3 kW receiver model. The model is used to predict the behavior of a receiver 100 kW of incident power which is the same of the current work. The same dimensions and the same operating parameters have been adopted (Table 1, Table 2).

• Case 2

The work of Pozivil et al [13,14] treated a 50 kW_th_ receiver model by numerical and experimental studies of conjugate transfers. The same main dimensions are renewed in our model. The value of the incident flux is considered in the resulting code of the algorithm solving the net flow method and is incorporated into ANSYS Fluent as UDF under boundary conditions. The calculation of dimensionless numbers makes it possible to make appropriate choices of the parameters of the CFD model. For a non-ideal compression and a perfect gas the inlet temperature is given by [14]:

(30) $$T_{in}=T_{amb}{\left(\frac{p_{in}}{p_{amb}}\right)}^{\frac{\gamma -1}{\gamma -\eta_{polytropic}}}$$

The same physical conditions are then repeated, namely the inlet flow, the geometrical parameters, the inlet temperature, the porosity of the absorber and the thermo-physical properties of the materials. Mass flow rates range from 20 to 160 kg/s.

#### Comparisons

The approximate trend of the air temperature outlet as a function of the air mass ﬂow rate is indicated by an exponential ﬁt in the Ref. [13].

$$T_{out}=632.2e^{-0.1067\dot{m}}+957.7e^{-0.01127\dot{m}}$$

With $R^{2}=0.9272$

**Fig. 5:**
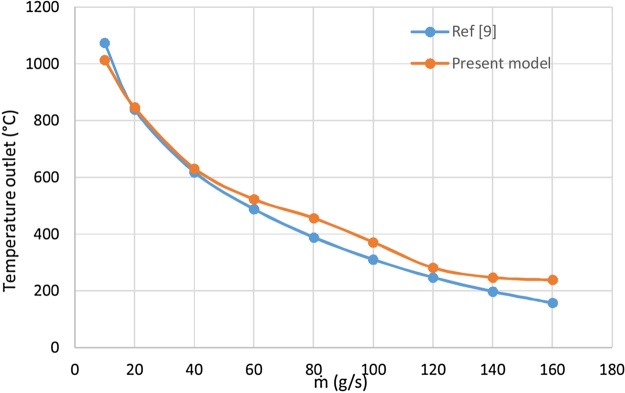
Comparison between the model results and those of the Ref. [13]: temperature outlet variation versus mass flow rate.

Fig. 5 shows the temperature outlet variation difference between the present model prediction and that of the Ref. [13] according to mass ﬂow rate.

The results show good agreement. The deviations may attribute to the value of the coefficient of determination.

The 50 kW model configuration giving the highest performance is chosen because this is the case where more information on the input and operating conditions is obtained (Table 3).

**Table 3:** Comparison between the model results and those of the Refs. [12,13].

		Thermal efficiencies (%)	Temperature outlet (K)
50 kW model	Ref. [13]	91.2	972
	Present model	92	976
100 kW model	Ref. [12]	90	1273
	Present model	92	1267

These comparisons have demonstrated that the numerical model developed is satisfactory for analyzing both the radiative transfers within the cavity and the heat transfer coupled with the air flow at the absorber.

### Receiver optimization methodology

The characteristics of the ANSYS Design Xplorer tool are used in this work. The optimization method proposed is the combination of the Kriging surface response method and MOGA. It enables optimization studies based on multiple objectives to take account of design uncertainties and to determine the best way to improve the reliability of the product. The framework generates a set of parameters (using Design of Experiments DOE) based on a mathematical sampling method. Output parameters are generated based on the parameter configured to construct response surfaces. Finally, using an optimization algorithm, optimal results are obtained on the generated response surfaces. The Response Surface (RSM) allows approximations via support points to represent the relationship between the output parameter and the input parameters (design parameters) based on regression methods [18,20]. Compared to the classic MOGA algorithm, the use of the Kriging meta-model allows a faster optimization process [20].

#### Kriging

Kriging is a meta-modeling algorithm that provides improved response quality and adapts to higher-order variations of the output parameter. This is a precise multidimensional interpolation combining a polynomial model similar to that of the standard response surface which provides a "global" model of the design space plus local deviations so that the Kriging model interpolates DOE points. The Kriging meta-model offers refinement features for continuous input parameters, including those with manufacturing values. The effectiveness of the Kriging algorithm is based on the ability of its internal error estimator to improve the quality of the response surface by generating refinement points and adding them to the areas of the response surface that have the most need to be improved.

#### MOGA

The Multi-Objective Genetic Algorithm (MOGA) is a variant of the most widely known NSGA-II (Non-Dominated Sorted Genetic Algorithm-II) based on controlled elitism concepts. It supports several objectives and constraints, and aims to find a global optimum. The MOGA is a powerful tool because it integrates global Pareto filters and leads to a Pareto global front. To do this, a hybrid variant of controlled elitism concepts, NSGA-II, is used. The Pareto regime rankings and objective manipulation and constraint are performed by a non-dominated fast sorting method which is an order of magnitude faster than traditional Pareto ranking methods. Genetic algorithms differ from more traditional optimization techniques to the extent that they involve a search for a "population" of solutions, not just one point. Each iteration or generation of a genetic algorithm involves a competitive selection that eliminates the wrong solutions. High-capability solutions are "recombined" with other solutions by exchanging parts of one solution with another. The solutions are also "mutated" by making a small change to a single element of the solution. Recombination and mutation are used to generate new solutions that are biased towards regions of space for which good solutions have already been seen [18,21]. The constraints are related to the limit values of the materials used.

#### Optimization model settings

Ninety auto-defined CCD samples were specified for the six independent parameters. For each of these specimens, an ANSYS Fluent simulation was performed and the outlet temperature and mass flow rate were extracted. A Kriging response surface was then constructed for each of the output parameters and combined as necessary for the objectives and constraints. The default value for automatic refinement of design points was chosen for Kriging regression. Finally, the following parameters of the MOGA method have been selected: "number of initial points" and "number of samples per iteration" have been set at 100, while the "maximum allowable percentage of Pareto" has been set at 70%. And the "maximum number of iterations" is set at 20. This means that optimization terminates its process when the resulting MOGA edge contains at least 70 points (70% of 100 in number of samples per iteration), or reaches the maximum number of iterations [18].

Multi-objective optimization using MOGA was conducted for a compromise between temperature outlet and mass flow as they are in conflict with each other. In practice, a set of optimal solutions for the design parameters is recommended so that the optimal solutions of Pareto can be selected by the designer according to the needs of the thermodynamic conversion system. The results made it possible to obtain the different combinations in order to have both exit temperatures and optimal energy quantities (Table 4).

**Table 4:** Candidate points giving ideal combinations for optimum temperatures and flows.

Candidates	Cavity thickness (m)	Insulating thickness (m)	porosity	Mass flow (kg/s)	Pressure (Pa)	Temperature outlet (K)	Thermal Efficiencies (%)
candidate 1 (Verified value)	0.019028	0.1023922	0.759473	0.1480	466555.06	1246.22 (1249.48)	95.12
candidate 2 (Verified value)	0.019054	0.1031376	0.744150	0.14501	348871.56	1248,85 (1251.14)	95.02
candidate 3 (Verified value)	0.019713	0.1038334	0.746211	0.14054	420869.17	1252,33 (1251.42)	95.07

## Conclusion

This work dealt with the numerical analysis of the radiative transfers of the cavity and the conjugate transfers of the absorber of a solar air receiver. Starting from this numerical model, a mathematical optimization combining a Kriging response surface and the MOGA was performed.

The following main conclusions can be drawn:

The coupling between the net-radiation method using the infinitesimal areas and the CFD code is interesting to model both the radiative transfers within the cavity and the conjugate transfers at the absorber level. This has allowed the hydrodynamic and thermal behavior of the receiver to be adequately predicted.

The Composite Center method allowed the generation of 90 design points for a plan of experiments. This constituted a database for creating a response surface using a regression analysis based on the Kriging method. The quantitative and qualitative analysis of the design parameters on the exit temperature showed that the porosity is the parameter having the most impact on the exit temperature with a percentage of 62%. The results of the multi-objective optimization made it possible to obtain 3 candidates giving the best combinations of design parameters from the fixed objectives which are to maximize both the temperature and the quantity of thermal energy at the output of the receiver. The best candidates from the compromise study for a global optimum and their validities were found and verified. Many studies investigate the influence of conception parameters individually. On the other hand this work presenting a flexible model allows the elaboration of ideal combinations for the fixed objectives. This makes it possible to adapt the receiver to any power or any output temperature of the desired fluid, thus allowing a better coupling between the receiver and the various systems of the plant.
